# Apparent Survival of Territorial Golden Eagles Using Non‐Invasive Genetic Profiling

**DOI:** 10.1002/ece3.72912

**Published:** 2026-01-08

**Authors:** D. Philip Whitfield, Brett K. Sandercock, Rob Ogden, Ruth Tingay, Patricia Whitfield

**Affiliations:** ^1^ Natural Research Brathens UK; ^2^ NINA Trondheim Norway; ^3^ Royal (Dick) School of Veterinary Studies and the Roslin Institute University of Edinburgh UK; ^4^ Wild Justice London UK

**Keywords:** *Aquila chrysaetos*, breeding dispersal, conservation, DNA profiling, eagle, life expectancy, mortality, population dynamics, raptor, Scotland

## Abstract

Conservation efforts for large raptors require robust estimates of fecundity, age at maturity and survivorship and population trajectories are often particularly sensitive to change in adult survival rates. Our study estimated apparent survival rates in territorial range‐holding Golden Eagles *Aquila chrysaetos* in Scotland, based on DNA extracted from moulted feathers to profile individuals genetically: the first study for this species using the method. Feathers were collected at or close to nest sites involving 104 territorial individuals, across 4 years (2006–2009), with repeat‐sampling in 21% of 442 occupied home ranges. Genetic profiles identifying individuals' detection/non‐detection were analysed to estimate annual apparent survival rates using Cormack–Jolly–Seber models. Our models included sex, as male survival was expected to be lower through greater male parental burdens, and two regions, the Outer Hebrides and the Inner Hebrides/Highlands, that host separate sub‐populations. Lower survival was expected in the Outer Hebridean archipelago through negative density‐dependence created by globally high densities in this isolated sub‐population. Estimates of male annual apparent survival rates (0.774–0.808) were markedly lower than females in both regions (0.878–0.882). Encounter rates of males (0.441–0.454) were also lower than females (0.639–0.754), probably because males spend less time near nest sites. Male life expectancy was ~50% lower in both study regions. We found no support for different regional survival. We recorded a few instances of breeding dispersal, mostly females moving to neighbouring territories. Illegal killing probably had negligible influence on apparent survival estimates, because the DNA method is not well‐suited for detecting persecution in Scotland. Our estimates of apparent survival were probably close to true natural survival. Annual rates for females were similar to previous estimates derived for the same ‘adult’ age/status class, which utilised other methods but did not separate the sexes. Sex difference in adult survival should be a feature of future studies of raptors and its role in demography and conservation.

## Graphical abstract

In a novel study for the species, DNA was extracted from moulted feathers over 4 years involving 21% of the Scottish population of territorial golden eagles, and individuals' genetic profiles were analysed for apparent survival rates. Results showed sexual differences but not between two sub‐populations; very few cases of breeding dispersal were documented. With a relatively high sampling effort our study allowed insights into the utility of this method and others that can estimate apparent survival of territorial eagles—an important metric in understanding their ecology.

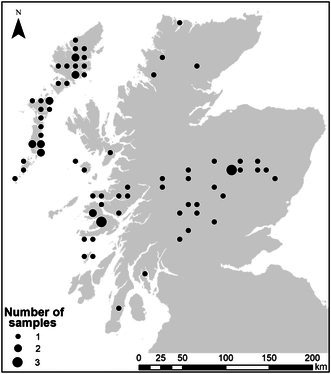

## 1 Introduction

Conservation of large raptors requires reliable information on fecundity, age at maturity and, especially, survival rates. However, it is difficult to estimate survival rates of large raptors after fledging. Challenges arise because birds occur at low density, with delayed maturity and lengthy duration of natal dispersal, and low reproductive rates, but with expected high survival for the relatively few reproductive products (Newton 1979; Sæther and Bakke 2000). Survival rates of fledged birds, particularly for older individuals, are important demographically because they primarily influence population persistence and change (Sæther and Bakke 2000; Whitfield et al. 2004; Penteriani et al. 2005; Monzón and Friedenberg 2018). Consequently, paradoxical to the challenge of their estimation, survival rates are critical to the modelling and conservation of large raptor populations.

Estimating survival rates requires longitudinal data from repeated sampling events of individually identifiable birds through ‘mark’ (or ‘capture’) followed by later detections during ‘recapture’ events. Estimation methods can be subject to potential biases, which for some can be more acute in large raptors because of difficulty in data collection. The various field methods used to underpin raptor survival estimation were reviewed by Newton et al. (2016) and marking techniques and analytical methods have both improved over time.

Several older field methods can be less robust such as use of metal ringing/banding data (Newton et al. 2016), since the ‘mark’ relies on observer ‘recaptures’. There can be biases in subsequent reporting of birds marked with metal rings, when physical recaptures of the marked bird and/or its ring/band are usually required to confirm identity. Increasing the ease of individuals' ‘recapture’ by making their identity more obvious to field observers using unique colour rings/bands and/or patagial wing tags can provide higher detection rates (Etheridge et al. 1997; Evans et al. 2009). Resighting methods still, nevertheless, depend on the underlying intensity and spatial focus of observer field efforts to provide recapture records.

More modern field methods involve marking birds with telemetry devices whereby identifying a marked bird to record ‘recaptures’ may be lessened or removed from the potential vagaries of field observer effort because the tracking device transmits information on location and status (Sergio et al. 2019). Early use of this technology involving VHF (radio) tags still required physical field searching efforts, with recaptures based on observers' use of receptor antennae (Kenward et al. 1999), and so this method again can have recapture uncertainty (Bunck et al. 1995). In spatially restricted study areas with intensive effort to monitor VHF‐tagged birds and their fates, radio telemetry can reveal information, which improves on earlier methods (Hunt et al. 1999, 2017). The most recent use of GPS‐telemetry tags to mark birds provides further advantages because the tags facilitate remote monitoring of birds' status, recapture and fate (USFWS 2016; Newton et al. 2016; Whitfield and Fielding 2017; Sergio et al. 2019), and thereby further removed from the potential biases of non‐random field search effort.

Use of individual genotyping from DNA samples to estimate apparent survival rates is a relatively novel method and was not reviewed in detail by Newton et al. (2016). Birds are ‘marked’ by their unique genetic profile derived from DNA samples, which can be obtained from non‐invasive sources such as moulted feathers at nest or roost sites (Rudnick et al. 2005; Horváth et al. 2005). An advantage of the genetic method, other than its potential non‐invasive feature, is that it allows monitoring of adult raptors that are otherwise difficult to trap and mark. Higher numbers of individual birds can be monitored through genetic profiling, which can improve accuracy and precision of estimates of demographic rates (Rudnick et al. 2008, 2009). Marking raptor nestlings by any method is much easier, and GPS‐tagging can provide more robust data without the biases associated with field recapture efforts but leads to right censorship of longitudinal data because of temporal limits determined by the technological lifespan of tags (Whitfield et al. 2022).

The genetic profile method can be used to explore several demographic and behavioural features (Waits and Paetkau 2005; Rudnick et al. 2008, 2009). For survival estimation, profiling is typically best employed for birds where the prospect of resampling the same individuals, or any replacements, is greatest through their fixation on the same spatial location repeatedly over time, such as a breeding home range or nest sites that can be re‐occupied each year (Rudnick et al. 2005; Booms et al. 2011; Vili et al. 2013; Bulut et al. 2016; Hou et al. 2018; Stokke et al. 2024). The method follows the detection or non‐detection of individuals identifiable by their genetic profile at specific locations, which limits the spatial prospect for ‘recapture’, rather than following the movements of birds individually ‘marked’ with tracking tags. In its non‐invasive form, by extracting DNA from moulted feathers to generate individual genetic profiles (Rudnick et al. 2005; Horváth et al. 2005; Waterlot et al. 2024), the method can be affected by imperfect detection because moulted feathers may not always be found for a territorial individual, DNA extractions may not be possible for some feathers, and PCR reactions may fail due to poor quality DNA samples (Lukacs and Burnham 2005; Vili et al. 2013; Gil‐Sánchez et al. 2021).

By sampling identifiable birds at specific locations, the genotype profile method can only estimate apparent survival, and not true survival and rarely for known age individuals because epigenetic ageing methods are available for few species of wildlife. The disappearance of an individual can have three causes (Sandercock 2020): (1) the individual has died, and apparent survival is a good index of true survival; (2) the individual has not died but moved to another unsampled location through breeding dispersal and apparent survival is lower than true survival (Greenwood 1980; Greenwood and Harvey 1982); and, (3) the individual has not died but has been usurped from the breeding home range by a competitor and re‐enters the more mobile non‐breeding floater component of the population, which can consequently have a lower probability of detection than territorial individuals (Hunt 1998; Hunt et al. 2017).

The contributions of breeding dispersal to variation in apparent survival can be examined by the DNA method if the sampling locations are within the limits of the breeding dispersal distance. In several large raptors, including eagles, breeding dispersal is rare, but when it occurs, the movements are typically at short distances to adjacent territories (Newton et al. 1989; Forero et al. 1999; Whitfield et al. 2009, 2022; Hernández‐Matías et al. 2010; Booms et al. 2011; Vili et al. 2013; Scherler et al. 2023; Väli and Rohtla 2025). However, by focusing on genetic profiles of birds at nest sites that are used persistently across years (Morollón et al. 2024), the DNA method will be less suitable for estimating apparent survival in raptors which show substantial mobility and frequent movements among intra‐ and inter‐annual breeding locations. High rates of breeding dispersal are typically associated with raptor species that track cycles (or ‘pulses’) of local abundance in their rodent prey (Therrien et al. 2014; Calladine et al. 2024).

Our study's aim was to estimate apparent survival rates in resident territorial range‐holding Golden Eagles *Aquila chrysaetos* in Scotland, based on DNA extracted from moulted feathers to profile individuals genetically. Feathers were collected at nest sites and their immediate environs within 93 home ranges involving 104 individuals profiled at least twice during a four‐year period, 2006–2009. Derived genetic profiles identified detection or non‐detection of individuals at home ranges which were then analysed with Cormack–Jolly–Seber mark‐recapture models to estimate annual apparent survival rates.

Our overarching objective was to obtain survival estimates essential for population modelling (Sæther and Bakke 2000; Whitfield et al. 2004; Penteriani et al. 2005; Monzón and Friedenberg 2018), but we also tested two hypotheses. First, we hypothesised a sex difference in adult apparent survival rates because male Golden Eagles have greater investment in food provisioning of their mate and nestlings during the breeding season and possibly, territory defence (Bergo 1987; Collopy 1984; Watson 2010; Ellis et al. 2024). Female investments in clutch production and incubation are relatively small compared to many other birds (Newton 1979; Watson 2010), and therefore, female survival should be higher.

Second, we hypothesised that apparent survival rates should differ between two regions of Scotland. Specifically, annual survival should be lower in the Outer Hebrides than in the rest of Scotland (here described as the Inner Hebrides/Highlands). The Outer Hebrides is an archipelago on the northwestern limits of Scotland that supports large numbers of Golden Eagles at particularly high densities (Eaton et al. 2007), which are higher than most parts of the globe (Ellis et al. 2024). This region is separated from the rest of Scotland by expanses of sea that Golden Eagles seem unwilling to cross (Fielding et al. 2024b) and the isolation is confirmed by genetic differentiation (Ogden et al. 2015) in accordance with a historical separation (Evans et al. 2013). Consistent, the home ranges of Golden Eagles in the Outer Hebrides are remarkably small (Fielding et al. 2024a). These considerations led us to expect, through higher density‐dependent pressures in a closed sub‐population through insularity, that survival rates of territorial occupants in the Outer Hebrides should be lower than in the rest of Scotland. Additionally, given the potential role of breeding dispersal in causing a difference between apparent survival and true survival (Sandercock 2020), we also evaluated the extent of year‐to‐year movements among territories within our study area.

Our final objective was to consider the possible influence that illegal persecution might have had on apparent survival estimates, through humans illicitly killing range‐holding Golden Eagles. Killing Golden Eagles is illegal in Scotland and hence the perpetrators try to hide their unlawful behaviour (Whitfield and Fielding 2017; RSPB 2024). The circumstances make it difficult to investigate or prosecute individuals in connection with a particular eagle death, especially when such criminality is conducted in remote rural areas. From multiple data sources involving several different species of raptors, it is nevertheless clear that illegal persecution is predominantly and repeatedly associated with landscapes dominated by intensive management for ‘driven shoots’ of Red Grouse *Lagopus scotica* (Gibbons et al. 1994; Whitfield et al. 2003, 2004, 2007; Amar et al. 2012; Ewing et al. 2023).

## 2 Methods

### 2.1 Feather Sampling

The sampling regime for feather collection and genetic profiling has been described previously by Ogden et al. (2015). Briefly, genetic samples were obtained by collection of moulted feathers, almost entirely by voluntary efforts of the Scottish Raptor Study Groups (SRSGs) membership. All feathers were collected from ranges considered to be occupied by a resident pair of eagles according to national survey criteria (Eaton et al. 2007). Non‐territorial birds may intrude into occupied ranges during their natal dispersal phase (Fielding et al. 2023). To minimise the prospect that non‐territorial birds were sampled, collectors were encouraged to collect feathers within the occupied territory at or close to the active nest site each year (Reid et al. 2019) and to record the distance of the feather(s) to the nest. All feathers used for genotyping purposes were collected at or within 200 m of the active nest, with 88% collected at or within 50 m of the active nest. Feathers were also collected during licenced visits to nests to ring nestlings or alternatively after a breeding attempt had failed or fledglings had left the vicinity of the nest site to avoid undue disturbance. Genotyped feathers were consequently collected between June and November.

Golden Eagles are socially monogamous and our assumption prior to analysing feathers from or near the nest site was that feathers came from a single pair of birds. Hence, all feathers were initially sexed and then two samples from a male and female (if both genders were detected at a nest site within a year) were then used for microsatellite DNA profiling. In practice, in seeking genotypes of both members of a mated pair, we often genotyped the same sex more than once in the same home range in a year (section ‘DNA analysis’ below).

**FIGURE 1:**
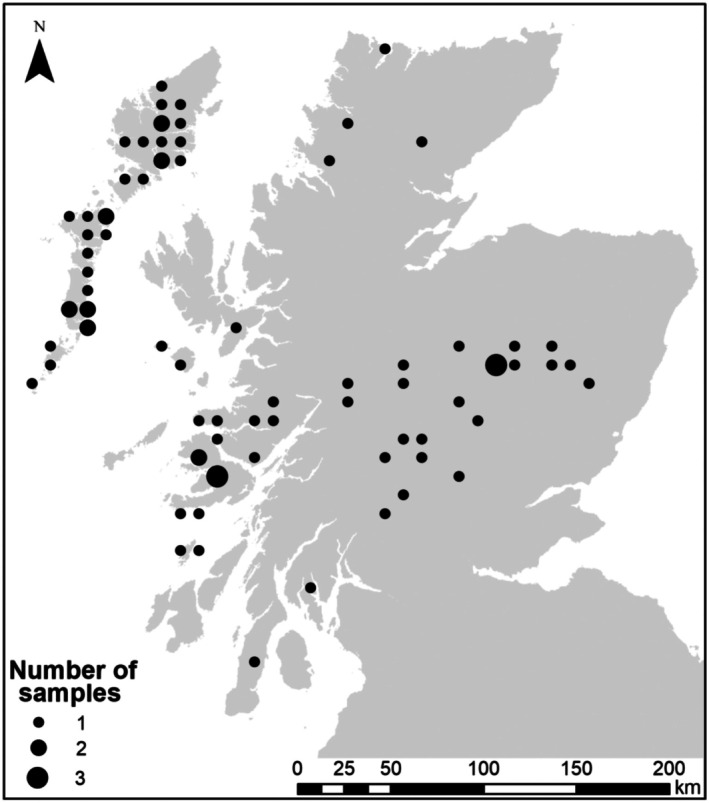
Numbers and distribution of Golden Eagle home ranges (by 10 km Ordnance Survey grid square) where genetic profiling of at least one sex was obtained from moulted feathers over at least two years 2006–2009: Number of samples = *n* home ranges by 10 km grid square. Two home ranges used in our study from the south of Scotland are not shown because of acute sensitivity regarding locations in this vulnerable region for the species. The Outer Hebrides region is the north‐to‐south archipelago in the northwest of the map. The Inner Hebrides/Highlands region is elsewhere in Scotland. Contains Ordnance Survey data Crown copyright and database right 2020.

Our study was based on home ranges where feather samples were collected in at least two years in the 4‐year period of 2006–2009 (Figure 1). In 93 ranges, at least one sex was sampled in more than one year, and so our study involved at least one sex in 21% of 442 occupied ranges in Scotland as censused in 2003 (Eaton et al. 2007).

Our monitoring involved 38 territories in the Outer Hebrides and 55 territories in the Inner Hebrides/Highlands (Figure 1). Within this monitoring, a total of 104 unique individuals were detected in the field study in at least 2 years 2006–2009, including 29 females and 13 males in the Outer Hebrides, and 44 females and 18 males in the Inner Hebrides/Highlands.

### 2.2 DNA Analysis

Our DNA extraction and analytical methods have been described in detail (Ogden et al. 2015). DNA was extracted from the blood spot within the superior umbilicus of the feather (Horváth et al. 2005). Downy feathers were not used to avoid potential confusion with feathers shed by chicks. All extractions were performed using the Qiagen DNeasy tissue kit (Qiagen GmbH, Hilden, Germany) following the manufacturer's instructions. DNA extracts were subjected to molecular sexing using a PCR test targeting the Z and W avian sex chromosomes of Golden Eagles (Ogden et al. 2015).

Male and female samples, from each territorial range per collection year, were genotyped using 10 microsatellite loci that were previously developed for other raptors (Martínez‐Cruz et al. 2002; Busch et al. 2005), but where successful cross‐species amplification has been confirmed for the Golden Eagle (Bourke and Dawson 2006; Dawnay et al. 2009). Individual profiles were created using the microsatellite markers amplified in two separate PCR reactions, optimised for degraded DNA samples. Multiplex 1 involved Aa02, Aa04, Aa27, Aa36, Aa39 (Martínez‐Cruz et al. 2002) and IEAAAG04 (Busch et al. 2005). Multiplex 2 contained Aa15, Aa26, Aa43 (Martínez‐Cruz et al. 2002) and IEAAAG15 (Busch et al. 2005). Both reactions were amplified under the same PCR conditions (Ogden et al. 2015).

Genotypes were scored by eye and multiple feathers from the same bird were matched using CERVUS 3.0 (Kalinowski et al. 2007). Tests of single‐sample replicate PCRs indicated that allelic dropout was not likely to be a widespread problem, but to control for possible effects of dropout, sample profiles that only differed due to loci displaying homozygote genotypes for different alleles were re‐genotyped and, where appropriate, a consensus genotype was formed for an individual. We repeated 112 identifications (*n* = 488 total profiles) by DNA extraction from more than one feather within a year by sex and home range (*n* = 376 count by range and sex) and recorded no inconsistency.

Of the individual profiles recovered, 68% of individuals displayed complete 10‐locus genotypes, with over 95% of birds having genotypes at eight loci or more. All 10 loci were successfully genotyped with a mean per locus genotyping rate of 95%, and all were polymorphic with an average of 5.5 alleles per locus (Ogden et al. 2015). The probability of observing two unrelated birds with the same genotype was estimated as *p* = 1.79 × 10^−6^ (mean polymorphism information content (PIC) across loci = 0.46). The probability of misidentification increased to *p* = 2.12 × 10^−5^ when assuming a relatively high degree of relatedness within the population and *p* = 2.95 × 10^−3^ for the probability of observing identical profiles if birds are all siblings, indicating that the risk of misassigning moulted feathers to individuals was remote (Ogden et al. 2015).

### 2.3 Mark‐Recapture Models

The genetic profiles based on microsatellite markers were used to create encounter histories for individual birds for the 4‐year period of 2006–2009. Each year within the encounter history was coded as 1 = detected with genetic methods or 0 = not detected during the breeding season. All birds included in the encounter histories were territorial pairs in occupied territories and were presumed to be at least four‐five years old (although see Whitfield et al. 2022). We analysed the encounter histories with Cormack–Jolly–Seber (CJS) models to estimate annual probabilities of apparent survival (*ϕ*) corrected for the probability of encounter (*p*).

Mark‐recapture analyses were conducted within an R environment with functions of the RMark package as an interface to Program Mark (White and Burnham 1999; Laake 2013). We selected factors to include in the global model for the probabilities of apparent survival (*ϕ*) and encounter (*p*). We modelled both parameters as a function of sex because females and males differ in reproductive roles and behaviour during the breeding season (Collopy 1984; Bergo 1987). Similarly, we modelled both parameters as a function of region because prior genetic analyses and movements of birds with GPS‐tags have shown that populations in the Outer Hebrides and the Inner Hebrides/Highlands are separated (Ogden et al. 2015; Fielding et al. 2024b). We explored time‐since‐marking models (tsm) in preliminary analyses but found no evidence that apparent survival in the interval after first encounter (*ϕ* ^1^) differed from later intervals (*ϕ* ^2+^, Sandercock 2020). We opted not to test models with effects of time‐dependence because we expected annual survival to be high and because the study duration was relatively short. Thus, our global model was a CJS model with the effects of sex and region in apparent survival, and in the probability of encounter: *ϕ* (sex × region), *p* (sex × region). We used the Fletcher's c‐hat procedure to evaluate the goodness‐of‐fit of the global model to the encounter histories and to calculate a variance inflation factor to correct for overdispersion ($\hat{c}$). Moderate amounts of overdispersion are common in mark‐recapture data and values of $\hat{c}$ < 3 indicate that the global model is an acceptable fit to the data.

We proceeded with model testing by fitting reduced models with fewer parameters. All models were constructed with design matrices and the logit‐link function. Our global model was relatively simple and we opted to test 25 candidate models with all possible combinations of a factorial model with an interaction term (sex × region), an additive model with main effects (sex + region), single factor models (sex or region) and a constant model (con). The parameter count (*K*) and the deviance were combined to calculate Akaike's Information Criterion (AIC) adjusted for small samples and overdispersion (QAIC*c*). Candidate models were ranked by the difference from the minimum AICc model (ΔQAICc) and model weights (*w*_*i*_) were used to determine the relative likelihood of a model within the set of candidate models. With 25 candidate models, we opted to use a threshold of QAICc < 5 as an arbitrary but reasonable threshold to bound presentation of the top models. The threshold resulted in models with a total weight sum (*wi*) = 0.967.

No single model received a majority of support, and several models were an equally parsimonious fit to the encounter histories. Thus, overall estimates of apparent survival and the probability of encounter were calculated by model‐averaging procedures. Estimates derived from each candidate model were weighted by the QAIC*c* weights (*wi*) and used to calculate a weighted mean where the unconditional SE was adjusted for model uncertainty. Life expectancy in years ($\hat{E}$) was calculated as a derived parameter from the expression $\hat{E}=-1/\ln\left(\hat{\phi }\right)$, where $\mathrm{SE}\left(\hat{E}\right)$ was calculated with an expression based on the delta method (Sandercock et al. 2022).

R code for the mark‐recapture models is provided in Appendix S1.

## 3 Results

### 3.1 Mark‐Recapture Models

Goodness‐of‐fit tests showed that our starting global model *ϕ*(sex × region), *p*(sex × region) was a good fit to the encounter histories with low levels of overdispersion (Fletcher's $\hat{c}$ = 1.134). We adjusted for overdispersion with the variance inflation factor and used QAIC*c* for model ranking and selection. No single model received a majority of support. Six candidate models were an equally parsimonious fit to the encounter histories (QAIC*c* ≤ 2, *wi* ≥ 0.06) and an additional 12 candidate models received weak support (QAIC*c* ≤ 5, *wi* ≤ 0.015, Table 1). The top models for apparent survival included single factor effects of constant, sex and region, whereas the best fit for the probability encounter was either an interactive model between sex and region or sex alone. Given that multiple candidate models received some support during model selection, we used model‐averaging procedures to calculate parameter estimates separately for each sex and region.

**TABLE 1:** Model selection for candidate models for apparent survival ($\hat{\phi }$) and encounter ($\hat{p}$) from non‐invasive monitoring of Golden Eagles in Scotland, 2006–2009.

Model structure	*K*	‐2LnL	QAIC*c*	ΔQAIC*c*	*wi*
*ϕ*(con), *p*(sex × region)	5	454.9	411.4	0.0	0.169
*ϕ*(con), *p*(sex)	3	460.6	412.3	0.9	0.108
*ϕ*(sex), *p*(sex × region)	6	453.6	412.4	1.0	0.104
*ϕ*(sex), *p*(sex)	4	459.3	413.2	1.8	0.068
*ϕ*(region), *p*(sex × region)	6	454.8	413.4	2.0	0.061
*ϕ*(con), *p*(sex + region)	4	459.6	413.4	2.0	0.061
*ϕ*(sex), *p*(sex + region)	5	457.5	413.7	2.3	0.055
*ϕ*(region), *p*(sex)	4	460.3	414.1	2.7	0.044
*ϕ*(sex + region), *p*(sex × region)	7	453.2	414.1	2.7	0.043
*ϕ*(sex + region), *p*(sex + region)	6	455.8	414.3	2.9	0.040
*ϕ*(sex + region), *p*(region)	5	458.4	414.5	3.1	0.036
*ϕ*(sex), *p*(region)	4	460.9	414.6	3.2	0.034
*ϕ*(sex × region), *p*(sex + region)	7	454.0	414.9	3.5	0.030
*ϕ*(sex × region), *p*(sex)	6	456.7	415.1	3.7	0.027
*ϕ*(sex + region), *p*(sex)	5	459.3	415.3	3.9	0.025
*ϕ*(sex), *p*(con)	3	464.0	415.3	3.9	0.024
*ϕ*(region), *p*(sex + region)	5	459.6	415.5	4.1	0.022
*ϕ*(sex × region), *p*(sex × region)	8	453.1	416.1	4.7	0.016

*Note:* Model factors included sex (female vs. male) and region (Outer Hebrides vs. Inner Hebrides/Highlands). Factors were combined in factorial models with interactions (×), additive models with main effects only (+), single factor models and intercept‐only models (con). Model fit was calculated from the parameter count (*K*), deviance (−2lnL) and quasi‐AICc values (QAIC*c*) corrected for small sample sizes and adjusted for overdispersion (Fletcher's $\hat{c}$ = 1.134). Models were ranked by ΔQAIC*c* and relative support for each model is given by the model weights (*wi*). Candidate models that received little support are omitted from the table (ΔQAIC*c* ≥ 5, *wi* ≤ 0.015).

### 3.2 Apparent Survival: Parameter Estimates

Based on the model‐averaging procedure, the annual probability of encounter ($\hat{p}$) was lowest for males in both regions (0.441–0.454), intermediate for females in the Inner Hebrides/Highlands (0.639), and highest for females in the Outer Hebrides (0.754) (Table 2).

**TABLE 2:** Annual estimates of apparent survival ($\hat{\phi }$) and the probability of encounter ($\hat{p}$) from non‐invasive monitoring of breeding Golden Eagles in Scotland, 2006–2009.

Parameter	Region	Sex	Estimate	SE	95% LCL	95% UCL
$\hat{\phi }$	Hebrides	Male	0.774	0.126	0.454	0.933
		Female	0.878	0.054	0.730	0.950
	Highlands	Male	0.808	0.102	0.536	0.939
		Female	0.882	0.058	0.716	0.957
$\hat{p}$	Hebrides	Male	0.454	0.152	0.200	0.734
		Female	0.754	0.082	0.563	0.880
	Highlands	Male	0.441	0.111	0.246	0.656
		Female	0.639	0.085	0.462	0.785
$\hat{E}$	Hebrides	Male	3.9	2.5	1.3	14.4
		Female	7.7	3.6	3.2	19.5
	Highlands	Male	4.7	2.8	1.6	15.9
		Female	8.0	4.2	3.0	22.8

*Note:* Estimates were calculated with model‐averaging procedures applied to the full set of candidate models. Future life expectancy in years ($\hat{E}$) was then calculated as a derived parameter from the expression $\hat{E}=-1/\ln\left(\hat{\phi }\right).$ Hebrides = Outer Hebrides, Highlands = Inner Hebrides/Highlands.

After correcting for the variation in imperfect detection, annual estimates of apparent survival ($\hat{\phi }$) were similar between the two regions but were lower among males (0.774–0.808) than females (0.878–0.882) (Table 2). Estimates of precision were better for the apparent survival of females than males due to a larger sample of birds and because females had a higher probability of encounter. Based on our estimates of apparent survival, the future life expectancy ($\hat{E}$) of a territorial eagle in Scotland was 4–5 years for males and 8 years for females (Table 2).

### 3.3 Breeding Dispersal and Persecution

Breeding eagles showed strong site fidelity, and most birds were only detected at a single territory (95%, 99 of 104). Nevertheless, we recorded five cases of breeding dispersal where eagles moved to a different territory between years. Four cases were females breeding in the Outer Hebrides (FX, HJ, GU and IR), and a single record was for a male in the Inner Hebrides/Highlands (BC) (Appendix S2). In the case of female IR, the bird moved back and forth between two territories in consecutive years. The unusual behaviour was supported not only by documentation of both territories being occupied by a pair, and with checks confirming no occupancy at alternative nest sites in the relevant years (Eaton et al. 2007), but also from genetic profiling of individuals in both territories. All documented cases of breeding dispersal were short‐distance movements to a neighbouring territory. Otherwise, it was not possible to determine if territory turnover was due to mortality or displacement.

Persecution was strongly suspected due to unusual behaviour and circumstantial records at three territories in the Highlands (A/GT56, D/P6, G/LB2), with confirmation that at least one other bird died of poisoning (female AK in 2007) (Appendix S2). It is very difficult to confirm criminal persecution legislatively (RSPB 2024). Our suspicions of persecution events at three territories were based on an observational history of high turnover with repeated annual presence of subadults, and a locational history of known or strongly implicated wildlife crime (e.g., Whitfield and Fielding 2017; RSPB 2024). We had no suspicions of a role for persecution in turnover in the other 89 territories monitored in our sample.

## 4 Discussion

In a novel monitoring program, we used repeated sampling of the genetic profiles of 104 territorial Golden Eagles to estimate apparent survival across 4 years. We also examined two hypotheses expecting lower male apparent survival, and lower apparent survival through negative density‐dependence in a sub‐population isolated at high‐density in an archipelago on the northwestern limits of Scotland (Outer Hebrides). Our research also examined the occurrence of breeding dispersal and its effects on apparent survival vs. true survival. We also considered the potential influence of illegal persecution on our apparent survival rate estimates in the context of our field and genetic profiling methodologies.

### 4.1 Apparent Survival Estimates

Our estimates of apparent annual survival for territorial females (0.88: Table 2) were similar to most other previous estimates for the same age class/breeding status (between 0.77 and 0.93), even if methods of estimation differed as did precision on true survival estimation (Table 3). Previous studies did not separate the sexes, and sex‐specific estimates were a new contribution from our study (but see Hunt et al. 2017). Sample sizes of marked birds were also relatively small in most previous studies: *n* = 16 (Crandall et al. 2019), *n* = 17 with 13 territorial (Harmata et al. 2018) and *n* = 26 (Tikkanen et al. 2024) but were substantial in one study, *n* = 536 (Millsap et al. 2022) although the sample size included all age classes (Table 3).

**TABLE 3:** Previously published estimates of Golden Eagle annual survival rates for adults and/or territorial range‐holders: Values in parentheses refer to 95% CI, unless otherwise stated, and combined both sexes in reporting. Method column refers to the field method used in data collection (sample size in parentheses). Comments column provides context on rates censoring for anthropogenic deaths (e.g., wind turbine collisions) and whether the study involved residents and/or migrants when migrants often have lower survival rates (Newton 1979, 2025). The results of the present study are also shown in the final four rows for ease of comparison, by region and sex (the accumulated sample size was 104).

Survival rate	Age class	Study area	Method	References	Comments
0.91 (0.86–0.96)	Breeding adults	California, USA	VHF‐telemetry (47)	Hunt et al. (2017)	Residents. Not censored for anthropogenic deaths (0.94 if so)
0.86 (0.60–1.00)	Adults	Montana, USA	GPS telemetry* (17)	Harmata et al. (2018)	Largely residents
0.93 (0.81–0.98)	Breeding adults	Montana, USA	GPS telemetry (16)	Crandall et al. (2019)	Residents
0.87 (0.84–0.89)	‘Adult’ (after year 3)	USA	GPS telemetry and ring/band returns**	USFWS (2016)	Resident and migratory birds. Not censored for anthropogenic deaths (0.93 if so)
0.90 (0.88–0.91)	‘Adult’ (after year 3)	Western USA	GPS telemetry and ring/band returns (536)**	Millsap et al. (2022)	Largely residents. Not censored for anthropogenic deaths
0.77 (range 0.62–1.0)	Breeding adults	Finland	DNA profiling of chicks, to infer parental turnover (201)	Kylmänen et al. (2023)	Largely residents
0.92 (0.84–0.97)	Breeding adults	Finland	GPS telemetry (26)	Tikkanen et al. (2024)	Residents
0.77 (0.45–0.93)	Breeding males	Outer Hebrides, Scotland	DNA profiling (13)	Present study	Residents
0.81 (0.53–0.94)	Breeding males	Inner Hebrides/Highlands, Scotland	DNA profiling (18)	Present study	Residents
0.88 (0.73–0.95)	Breeding females	Outer Hebrides, Scotland	DNA profiling (29)	Present study	Residents
0.88 (0.72–0.96)	Breeding females	Inner Hebrides/Highlands, Scotland	DNA profiling (44)	Present study	Residents

* Tail‐mounted tags with limited duration.

** Sample size unclear for the ‘adult’ class.

To our knowledge, there are no previous estimates of apparent survival for Golden Eagles based on genotyping of resident territory occupants. A French study provided useful insights from territorial occupants on sampling efforts required to meet detection of survival rate change, but longitudinal samples across sufficient territories were inadequate to provide an estimate of apparent survival (Waterlot et al. 2024). Research on Finnish Golden Eagles involved a genetic profiling method from DNA samples obtained from feathers of both territory holders and nestlings (Kylmänen et al. 2023). Turnover rate of territorial (range‐holding) birds was estimated using relatedness between chicks born in the same territory but in different years. The estimated turnover rate of territory holders was 0.23, implying an apparent survival rate of 0.77 for adult eagles of both sexes (Table 3).

Territorial parents were also sampled, but Kylmänen et al. (2023) did not use these data to validate or cross‐check estimates from the chosen method utilising nestlings' genetic profiles. Their estimate of apparent survival rate was lower than other estimates for the same age class, including those of the present study, but most of those estimates are probably closer to true survival rates (Table 3). More specifically, estimates of apparent survival based on genetic profiling are also substantially lower than an estimate for territory holders (0.92: Table 3) derived from GPS telemetry elsewhere in Finland (Tikkanen et al. 2024). The authors remarked that the difference ‘likely reflects additional factors such as site fidelity’. However, ‘site fidelity’ is a term for the trait contrary to breeding dispersal and many studies indicate breeding dispersal is typically not common enough in most large raptors to provide a necessary explanation for the difference (including the Golden Eagle, as in the present study).

Previous studies using genetic profile methods similar to our approach have involved the closely related Eastern Imperial Eagle ( *Aquila heliaca* ) with estimated apparent survival rates for territory holders of 0.84 in Kazakhstan (Rudnick et al. 2005) and 0.65–0.72 in Hungary (Vili et al. 2013). The Hungarian population was expanding, despite the relatively low apparent survival rate of breeders. Both populations of Eastern Imperial Eagles are migratory, and migratory populations of Golden Eagles may also have lower survival rates (Millsap et al. 2022; Newton 2025). While not directly comparable to the adult age classes, survival estimates for Eastern Imperial Eagles from Bulgaria using Argos/GPS telemetry were 0.80 for third calendar year birds (Stoychev et al. 2014).

Genetic profiling has also been used in an Estonian study to estimate apparent survival of breeding birds in the migratory Lesser Spotted Eagle *Aquila pomarina* (Väli and Rohtla 2025). The authors reported an annual rate of 0.81 (both sexes) and, with typically large overlapping confidence limits, this was not significantly lower than independent estimates from colour‐ringing (0.88) and GPS telemetry (0.84).

### 4.2 Sex Difference in Survival

We hypothesised that male Golden Eagles should have lower apparent survival. Males were less likely to be encountered in our sampling (see also Vili et al. 2013; Waterlot et al. 2024). Sex differences in detection were likely due to the differing parental roles and the time each sex spent at or near the nest, where feather collections were deliberately focussed to avoid the possibility of sampling intruders.

In addition, we found support for a sex difference in demography with lower estimates for apparent survival in males, with relatively substantial effects on life expectancy. Male Golden Eagles take the burden of food provisioning of their mate and nestlings during the breeding season and possibly, territory defence (Bergo 1987; Collopy 1984; Watson 2010; Ellis et al. 2024). Hence, it may be expected that male survival should be lower. Parental role division in another large raptor may possibly explain the more pronounced senescence in fecundity for male White‐tailed Eagles ( *Haliaeetus albicilla* ) (Murgatroyd et al. 2018). A review of raptor survival rates nevertheless concluded that gender differences were not statistically significant (Newton et al. 2016), even if several studies have reported slightly lower survival rates for males, across different methods (e.g., Väli and Rohtla 2025). Most previous studies of adult Golden Eagle survival have not previously reported or considered sex differences in survivorship (Table 3).

Our finding of lower male survival may have biological credence when the life expectancy consequences were marked with 50% lower male life expectancy in both of our study regions (Table 2). Precision was low with large overlap between the sexes in the 95% CI for life expectancy. Nonetheless, attribution of differences by statistical significance in any feature of survivorship in large raptors, when survival rates are fundamentally high, requires substantial sampling effort (Waterlot et al. 2024). Practically, such effort, to achieve sufficient statistical power, may often be difficult or impossible to achieve for species that occur at low densities with high demands on supporting fieldwork.

In interpreting our results, however, we suggest that greater credence should be given to our estimates from females because: (1) the sampling of females was better, as shown by the higher probability of encounter and lower variation around estimates (Table 2), due to feather collections being centred at nest sites, where through parental role division females spend more time (Vili et al. 2013; Waterlot et al. 2024) and; (2) in Golden Eagles the female is assumed to be the limiting sex demographically (Hunt et al. 2017).

The assumption of females as the limiting sex comes from an intensive Californian study involving VHF‐tagged birds in which more males were encountered across several age classes, including birds in nests, and this sex ratio bias (~62%–64% males) was similarly reflected in estimated survival rates across combined post‐fledging age classes (Hunt et al. 2017). Hence, Hunt et al. (2017) reasonably did not examine any sex difference in adult survival (Table 3). Research on GPS‐tagged Golden Eagles hinted that Alaskan males could be more likely to be floaters (Lewis et al. 2025), which is consistent with the earlier conclusion of females being the limiting sex in California.

Alternatively, however, if territorial males may have lower life expectancy (as suggested by our study) the sex difference could lead to an opposite bias in sex ratio, at least in the availability of adult males and may make males the limiting sex in a species where biparental care is obligate and monogamy appears invariable (Ellis et al. 2024). Other results from Scotland (*cf*. Hunt et al. 2017) showed no sex ratio bias in nestlings at the age when tracking tags were deployed (Whitfield and Fielding 2017). Moreover, male Scottish Golden Eagles do not secure a territorial occupancy when younger (Whitfield et al. 2022) and so our finding of lower male survival in territorial birds seems unlikely to be due to being younger. Sex ratio bias in Golden Eagles and related species has been studied previously (Edwards et al. 1988; Bortolotti 1989; Ferrer et al. 2009; Katzner et al. 2014) but more comparative estimates are needed and, particularly, on sex differences in survival.

### 4.3 Regional Difference in Survival

We hypothesised survival rates should be lower in the Outer Hebridean sub‐population than elsewhere in Scotland. Our expectation was based on previous observations of marine movement barriers (Fielding et al. 2024b), genetic differentiation (Ogden et al. 2015), historical separation (Evans et al. 2013) and regional differences in breeding densities (Eaton et al. 2007; Fielding et al. 2024a). These features imply expectation of negative density‐dependent effects on demographic metrics that may include a reduction in adult survival.

The hypothesis of sub‐population differences was rejected because we found no evidence of a regional survival difference in either sex. The expectation of negative density‐dependent effects may be more apparent in other demographic metrics such as breeding productivity (Eaton et al. 2007; Whitfield et al. 2007) or age of territory settlement (Whitfield et al. 2022).

### 4.4 Influence of Breeding Dispersal and Usurpation From Territories

We recorded a few instances of breeding dispersal which, being detected, did not reduce our estimates of apparent survival. Four of five records involved movements of females, and all were to neighbouring territories. Our findings confirmed three expectations from previous research: breeding dispersal in large raptors is rare (Forero et al. 1999; Whitfield et al. 2009; Hernández‐Matías et al. 2010; Booms et al. 2011; Vili et al. 2013; Väli and Rohtla 2025); when breeding dispersal occurs it is more likely in females (Whitfield et al. 2009); and any movements are typically to neighbouring territories (Booms et al. 2011).

A greater likelihood of female movement agrees with other features of dispersal (Greenwood and Harvey 1982; Whitfield et al. 2024). The restriction of rare shifts to neighbouring territories is consistent with territorial Golden Eagles maintaining a balance between retention of their settled range while also exploring potentially better options. The balance seems manifested by periodic excursions away from their home range, which while probably a universal trait, are short in duration and spatially limited (Watson et al. 2014; Whitfield et al. 2022). Hence, and as some surveillance of neighbouring territories and their occupancy status can probably be achieved at territorial boundaries, it is not surprising that the rare breeding dispersal events are to neighbouring territories.

An additional mechanism that might cause apparent survival to be lower than true survival, is if a bird's disappearance from a sampling regime based on territorial range occupants was not through death but because it was usurped as an occupant by a competitor. Such birds could re‐enter the floating component of the population but would not be ‘recaptured’ under a genetic profiling method devoted exclusively to sampling home range occupants.

Territory usurpation is seldom considered explicitly when evaluating the accuracy of apparent survival in describing true survival (Väli and Rohtla 2025). The DNA‐based sampling methods of our study cannot be used to investigate usurpation. However, from previous research on Scottish Golden Eagles marked with GPS‐tags, range abandonment following usurpation by a competitor appears to be a rare event. Fielding et al. (2024a) had location data from 104 GPS‐tagged eagles that were range‐holders, including 69 with more than 1 year of post‐settlement data. There was only a single record of range abandonment and re‐settlement elsewhere, which was probably because the bird's original partner had been illegally killed (E. Weston, personal communication; D.P. Whitfield et al., unpublished data). The same dataset, on the other hand, included several examples where recovery of dead eagles marked with GPS‐telemetry tags and subsequent autopsy of the dead birds suggested an ‘eagle‐on‐eagle’ cause of death, implicating natural but fatal interactions during takeover events (D.P. Whitfield et al., unpublished data). Similarly, a recent study of Golden Eagles in Alaska reported nine transitions between territorial and floater status for birds marked with GPS‐tags. All transitions were floaters settling to become territorial holders, with no examples of territorial birds returning to become floaters (Lewis et al. 2025).

### 4.5 Influence of Persecution

Considerable research involving several raptors, including Golden Eagle consistently shows that the main perpetrators of illegal persecution are involved in the intensive management of Red Grouse (Gibbons et al. 1994; Whitfield et al. 2003, 2004, 2007; Amar et al. 2012; Ewing et al. 2023). Culling of predators is part of management to create very high grouse densities as deemed necessary for ‘driven shoots’, a sporting practice unique to the UK (Thompson et al. 2009, 2016). In Scotland, this land management practice is geographically restricted, predominantly in the eastern and central Highlands (Whitfield et al. 2003; Smart et al. 2010; Murgatroyd et al. 2019; Ewing et al. 2023).

Illegal persecution, largely associated with intensive management for Red Grouse, was evidentially strongly implicated in markedly lowering the survival rates of Scottish Golden Eagles satellite tagged as nestlings (Whitfield and Fielding 2017). An increased mortality rate was largely (but not exclusively) attributed to the sudden cessation of tag records. The events were not due to technological failure but were associated with failures to recover tags or carcasses despite intensive searches and were concentrated in and around areas managed intensively for Red Grouse.

Four birds' disappearance from our sampling regime (from a total of 104 individuals and much fewer ‘disappearances/deaths’: Appendix S2) were known or strongly suspected to be due to illegal killing by humans. The three suspicious disappearances coincided with locales identified by other means as places known for illegal killing of Golden Eagles and other raptors (Whitfield and Fielding 2017; Murgatroyd et al. 2019; Newton 2021; Ewing et al. 2023). Persecution, therefore, probably slightly reduced our apparent survival estimates for territorial range‐holding occupants in the Highlands. The possible effects were likely minimal given the sampling effort for this region where persecution of territorial occupants was not suspected in the large majority of sampled territories.

While we were confident that persecution had negligible effect on our survival estimates, this should not infer that persecution is not influential in reducing Golden Eagle survival in and around ‘Red Grouse moors’ in parts of Scotland (Whitfield and Fielding 2017). The substantial reduction in survival of GPS‐tagged eagles previously documented by Whitfield and Fielding (2017) was mainly based on younger age classes of birds. GPS‐tagging monitors individual birds and their fate whereas the DNA profiling method as used in our study monitors adult birds around nest sites. The DNA profiling method consequently will not be useful to detect losses to persecution if young birds are killed before they can settle, nest build and thereby moult feathers at nest sites that can be collected for genetic profiling (Whitfield and Fielding 2017; RSPB 2024).

## 5 Conclusions

Using genetic profiling of individual Golden Eagles from DNA extracted from moulted feathers we produced novel estimates of apparent survival for territorial occupants in Scotland. Estimates were comparable with published estimates derived by other methods for the same species (Table 3), and for other related species using the same non‐invasive DNA method (Rudnick et al. 2005; Väli and Rohtla 2025). We recorded a few instances of breeding dispersal, mostly by females moving to neighbouring territories, confirming that in our study species and related species it is unusual and spatially restricted (Whitfield et al. 2009; Vili et al. 2013; Fielding et al. 2024a). Occupation of a breeding territory in Golden eagles seems predominantly a ‘decision for life’ (Whitfield et al. 2022).

Our monitoring effort allowed testing for sex differences for which, as hypothesised, there was evidence of markedly lower male survival. A sex difference was found in both of our study regions that support separate sub‐populations. With the exception of Hunt et al. (2017), the possibility of sex differences in survival has not been formally considered before in Golden Eagles (Table 3). This shortage of consideration may be through a combination of not expecting a sex difference (Newton et al. 2016) and the difficult need for large sample sizes to reveal it, if it exists. Hunt et al. (2017) provided reasoned arguments as to why their Californian research did not formally examine a sex difference in survival. However, our finding of a marked sex difference in life expectancy of territorial birds in Scotland has revealed interesting geographical variation and more comparative data are needed.

## Author Contributions

**D. Philip Whitfield:** conceptualization (lead), data curation (supporting), formal analysis (equal), funding acquisition (lead), investigation (equal), methodology (equal), project administration (lead), resources (lead), supervision (lead), writing – original draft (lead), writing – review and editing (equal). **Brett K. Sandercock:** data curation (supporting), formal analysis (equal), investigation (equal), methodology (equal), validation (equal), writing – original draft (lead), writing – review and editing (equal). **Rob Ogden:** data curation (supporting), formal analysis (equal), investigation (supporting), methodology (supporting), validation (equal), writing – review and editing (equal). **Ruth Tingay:** conceptualization (lead), data curation (supporting), project administration (supporting), writing – review and editing (equal). **Patricia Whitfield:** data curation (lead), funding acquisition (supporting), project administration (supporting), writing – review and editing (equal).

## Funding

Funding was from Natural Research. B.K.S. was supported by basic funding to the Norwegian Institute for Nature Research from the Research Council of Norway (Project No. 160022/F40).

## Ethics Statement

Ethical review and approval were not required for our study given the non‐invasive nature of our basic method. Nests were visited for feather collection under appropriate licences for permission of disturbance, and if needed, handling, ringing and tagging from the relevant authority managed by Scottish Natural Heritage and British Trust for Ornithology. Licences were granted to a large number of individuals who were almost entirely SRSG volunteers under the Wildlife and Countryside Act 1981 (as amended). Our study used moulted feathers and collection of samples did not require live capture or handling of birds.

## Conflicts of Interest

The authors declare no conflicts of interest.

## Supporting information


**Appendix S1:** ece372912‐sup‐0001‐AppendixA.R.

**Appendix S2:** ece372912‐sup‐0002‐AppendixB.csv.

## Data Availability

We cannot provide the exact locations of the feather collection sites because disclosure would publicise nest sites for a protected species, which are protected under UK legislation. Approximate locations are given for collection sites in Figure 1. R code for mark‐recapture analyses is in Appendix S1, and the raw data by coded territory and coded individual eagle identities are in Appendix S2.
